# Adenovirus-mediated small interfering RNA targeting ezrin induces apoptosis and inhibits metastasis of human osteosarcoma MG-63 cells

**DOI:** 10.1042/BSR20180351

**Published:** 2018-08-29

**Authors:** Zhi-Wei Tao, Ping-An Zou

**Affiliations:** Bone and Soft Tissue Sarcoma Department, Jiangxi Cancer Hospital, Nanchang 330029, P.R. China

**Keywords:** Adenovirus, Apoptosis, Ezrin, Invasion, MG-63 cells, Migration, Osteosarcoma, Proliferation, Small interfering RNA

## Abstract

Osteosarcoma is a disease prone to recurrence and metastasis, and adenovirus expression vector is frequently studied as a therapeutic target of osteosarcoma in recent years. The present study attempts to explore the effect of adenovirus-mediated siRNA targetting ezrin on the proliferation, migration, invasion, and apoptosis of human osteosarcoma MG-63 cells. Human osteosarcoma MG-63 cell line was selected for construction of recombinant adenovirus vector. The mRNA and protein levels of ezrin, Bcl2-associated X protein (Bax), B cell lymphoma-2 (Bcl-2), p21, p53, Caspase-3, matrix metalloproteinase (MMP) 2 (MMP-2) and MMP-9, Cyclin D1, and cyclin-dependent kinase 4a (CDK4a) were determined. Through ELISA, the levels of Caspase-3, MMP-2 and MMP-9 were examined. Finally, human osteosarcoma MG-63 cell viability, growth, invasion, migration, and apoptosis were detected. Initially, adenovirus expression vector of ezrin was constructed by ezrin 2 siRNA sequence. Adenovirus-mediated siRNA targetting ezrin reduced expression of ezrin in MG-63 cells. The results revealed that adenovirus-mediated siRNA targetting ezrin elevated expression levels of Bax, p21, p53, and Caspase-3, Cyclin D1, and CDK4a and reduced expression levels of Bcl-2, MMP-2 and MMP-9. Furthermore, adenovirus-mediated siRNA targetting ezrin inhibited human osteosarcoma MG-63 cell viability, growth, invasion, and migration, and promoted apoptosis. Our study demonstrates that adenovirus-mediated siRNA targetting ezrin can induce apoptosis and inhibit the proliferation, migration, and invasion of human osteosarcoma MG-63 cells.

## Introduction

Osteosarcoma is a kind of cancerous tumor in the bone, which is the commonest primary bone cancer [1,2]. Osteosarcoma occurs most widely amongst children and adolescents [3]. Osteosarcoma is hardly ever diagnosed before the age of 5 while its incidence increases significantly by the time of puberty, with its incidence peak during the teen years and the second peak occurs in the elderly (aged ≥60 years) [4]. Its occurrence is mainly influenced by sex, age, ethnicity, tumor size, genetic factors, and environmental insults [5]. It frequently occurs in other cancer patients after therapeutic radiation, in patients with some kind of cancer predisposition syndromes, as well as in individuals with Paget disease of the bone [6]. Osteosarcoma is a highly metastatic, aggressive, and lytic tumor and the human lung is a frequent metastatic site for osteosarcoma [7]. The long-term survival rate in patients with osteosarcoma receiving surgical intervention is estimated to be 20–30% [8]. Although adjuvant chemotherapy has greatly improved the outcome of osteosarcoma to a certain degree, it remains practically the same during the past 30 years, possibly due to resistance to chemotherapies [9]. New treatment strategies for osteosarcoma are in urgent need [10].

Ezrin, also known as cytovillin or p81 protein, is an actin-binding protein in humans and a member of the ezrin, radixin, and moesin (ERM) family of proteins, which contribute to stabilizing the plasma membrane-formed structures [11]. It is one component part of cell-surface structures that participates in the adhesion to extracellular matrix, and it has been suggested in membrane–cytoskeleton interactions [12]. Meanwhile, it also plays a significant role in various cellular processes, including cell migration, formation, adhesion, and signaling pathways [13]. The correlations between ezrin expression and malignant tumors have been revealed amongst multiple human cancers, including prostate cancer, colorectal cancer, and osteosarcoma [14–16]. A previous study reported that mRNA expression of ezrin may serve as a predictor and a prognostic factor of potential metastasis in lung amongst Chinese osteosarcoma patients [17]. Recently, small molecules that are able to prevent the metastasis of osteosarcoma cells by targetting ezrin is discovered, making it requisite to explore the predictive role of ezrin in osteosarcoma [18]. The objective of the present study, therefore, is to determine whether the carrier for recombinant adenovirus targetted ezrin affects the cell viability of human osteosarcoma MG-63 cells. We hypothesized that adenovirus-mediated siRNA targetting ezrin may inhibit the proliferation, metastasis, and invasion and promote apoptosis of osteosarcoma MG-63 cells, with hope to be helpful for future treatment of osteosarcoma.

## Materials and methods

### Cell culture

Human osteosarcoma MG-63 cell line was obtained from the laboratory of Jiangxi Cancer Hospital and stored in liquid nitrogen. The cells were instantly dissolved at 37°C and then centrifuged at 1000 rpm for 5 min to remove the upper cryopreservation medium. MG-63 cells were re-suspended in Dulbecco’s modified Eagle’s medium (DMEM, Gibco, Life Technologies, Darmstadt, Germany) containing 10% FBS and then cultured in an incubator (Heraeus Holding GmbH, Hanau, Germany) with 5% CO_2_ at 37°C, with medium changed once every 3 days. When growing to 80% confluence, the cells were digested by 0.25% trypsase (1.5 ml) and subcultured at a ratio of 1:4. When growing to logarithmic growth phase and separation from the culture medium, MG-63 cells were digested by 0.25% trypsase (1.5 ml) for several minutes, and then added with 4.5 ml DMEM (Gibco, Life Technologies, Darmstadt, Germany) containing 10% FBS to stop digestion when the cells were observed retracted and rounded under a microscope. The cells were triturated into single cell suspension, and then centrifuged. With supernatant discarded, the cells were counted in the counting plate after washing with PBS (4.0 ml) for three times, and then the concentration was adjusted based on operation requirements.

### Construction of recombinant adenovirus vector

The sequence of human full-length *ezrin* gene was obtained from GenBank. Four recombinant adenoviruses that expressed siRNA targetting ezrin were designed (Table 1), and synthesized by Shanghai GenePharma Co., Ltd. (Shanghai, China). The most suitable siRNA was selected as the target sequence. Gene fragments were synthesized *in vitro*, containing *Bacillus amyloliquefaciens* H I (BamHI) and HindIII restriction enzyme cutting sites, as well as 5′-ATGCTATGTTGGAATACCTTTCAAGAGA-AGGTATTCCAACATAGCAT-3′ hairpin-like dsDNA structures. The gene fragments were inserted into pSIlence 2.1 neo vector, transfected with *Escherichia coli* DH5a, and then detected using reverse-transcription quantitative PCR (RT-qPCR), double enzyme digestion and sequencing. The fragments were then observed with a fluorescence microscope (Nikon, Tokyo, Japan) for 3–5 days. After transfecting with Lipofectmaine™ 2000 (Invitrogen, Carlsbad, CA, U.S.A.) and selected by 400 μg/ml G418, the siRNA transfected ezrin (si-ezrin) was subcultured at a ratio of 1:10 till stable si-ezrin recombinant adenovirus plasmids were obtained. DH5a cells at logarithmic growth phase were digested by trypsase, adjusted to a concentration of 10^5^/ml, and seeded in a 96-well plate (100 μl/well). Adenovirus vector and si-ezrin recombinant plasmids were collected after 24 h, seeded after 10^−4^, 10^−5^, 10^−6^, 10^−7^, and 10^−8^ dilution, respectively, with three wells per group. The plasmids were cultivated in an incubator (Heraeus Holding GmbH, Hanau, Germany) (37°C, 5% CO_2_) for 18 h, and then counted with the application of the fluorescence microscope to calculate the virus titer. Virus titer (pfu/ml) = (10 × mean fluorescence intensity)/corresponding dilution.

**Table 1:** The siRNA sequences for ezrin 1, ezrin 2, ezrin 3, and ezrin 4

Target gene	Sequence
*Ezrin 1*	5′-GGAAUCCUUAGCGAUGAGA-3′
	5′-CCUUAGGAAUCGCUACUCU-3′
*Ezrin 2*	5′-AUGCUAUGUUGGAAUACCU-3′
	5′-UACGAUACAACCUUAUGGA-3′
*Ezrin 3*	5′-GAAUCCUUAGCGAUGAGAU-3′
	5′-CUUAGGAAUCGCUACUCUA-3′
*Ezrin 4*	5′-AUGUCCGAGUUACCACCAU-3′
	5′-UACAGGCUCAAUGGUGGUA-3′
Control	5′-GACUUCAUAAGGCGCAUGC-3′
	5′-CUGAAGUAUUCCGCGUACG-3′

### Cell transfection and grouping

The human osteosarcoma MG-63 cells were seeded into a 24-well plate (0.5 × 10^5^ cells/well), and cultured at 37°C with 5% CO_2_. When growing to 80% confluence, the MG-63 cells at logarithmic growth phase were transfected with Lipofectamine™ 2000 (Invitrogen, Carlsbad, CA, U.S.A.) and assigned into the control, (added with 0.1 mol/l PBS, 100 μl), empty vector, (transfected with 100 mol empty vector plasmid), and test groups (transfected with 100 mol si-ezrin recombinant adenovirus plasmid). Total RNA were extracted by TRIzol (Sangon Biotech Co., Ltd., Shanghai, China) for RT-qPCR 48 h after transfection. The total protein was collected by BCA assay for Western blot analysis to select the most suitable siRNA.

### RT-qPCR

After transfection for 48 h, the total RNA was extracted with the use of TRIzol (Sangon Biotech Co., Ltd, Shanghai, China) in accordance with the manufacturer’s instructions, and then total RNA was reverse transcribed into cDNA according to the following steps. Total RNA (10 μl) were mixed with 50 μmol/l Oligo-dT (1 μl) and diethyl pyrocarbonate (DEPC) (1 μl), reacted at 70°C for 10 min, and then quickly cooled on ice for 2 min. The solution was added with 5× reverse transcription (RT) buffer (4 μl), 0.1 mol/l dl-DTT (2 μl), dNTPs (1 μl), RNase inhibitors (1 μl), SuperScript™ RT II (0.5 μl), and 1% DEPC water (0.5 μl) in turn, mixed and reacted at 42°C for 52 min, at 70°C for 15 min, and then cooled on ice to stop the reaction. The mixture was added with 80 μl sterile water and stored in refrigerator at −20°C. Using cDNA as template, the primers for RT-qPCR were as follows: ezrin, Bcl2-associated X protein (Bax), B cell lymphoma-2 (Bcl-2), p21, p53, Caspase-3, matrix metalloproteinase (MMP) 2 (MMP-2) and MMP-9 (Table 2). The PCR included cDNA (10 μl), PCR buffer (2×) (25 μl), forward and reverse primers of ezrin (recombinant ezrin based on the siRNA of ezrin 2) (1 μl for each) (25 μM), ezrin probe (1 μl), human glyceraldehyde-3-phosphate dehydrogenase (GAPDH) (20×) (2.5 μl), and then double distilled water (ddH_2_O) was added to reach a total volume of 50 μl. The RT-qPCR condition was 1 cycle of predenaturation at 95°C for 2 min, 40 cycles of denaturation at 94°C for 50 s, annealing at 59°C for 50 s, and extension at 72°C for 60 s. The mRNA expression of each gene was detected with GAPDH as the internal reference. The experiment was repeated three times.

**Table 2:** Primer sequences of ezrin, Bax, Bcl-2, p21, p53, Caspase-3, MMP-2, MMP-9, and GAPDH for RT-qPCR

Target gene	Sequence
*Ezrin*	5′-CCCTCCAGTTCAAGT-3′
	5′-AAGCCAAAGGTCTGT-3′
*Bax*	5′-ACACCTGAGCTGACCTTGGA-3′
	5′-CCGTGTCCACGTCAGCAATC-3′
*Bcl-2*	5′-TGCGCTCAGCCCTGTG-3′
	5′-GGTAGCGACGAGAGAAGTCATC-3′
*P21*	5′-GCGCCATGTCAGAACCGGCTG-3′
	5′-TCCTCCCAACTCATCCCGGCC-3′
*P53*	5′-GTACCGTATGAGCCACCTGAG-3′
	5′-CGTCCCAGAAGATTCCCAC-3′
*Caspase-3*	5′-AGAGCTGGACTGCGGTATTGAG-3′
	5′-GAACCATGACCCGTCCCTTG-3′
*MMP-2*	5′-GGAATGCCATCCCTGATAACCT-3′
	5′-TTCCAAACTTCACGCTCTTGAGA-3′
*MMP-9*	5′-GGAGACCTGAGAACCAATCTC-3′
	5′-TCCAATAGGTGATGTTGTCGT-3′
*GAPDH*	5′-GAAGGTGAAGGTCGGAGTC-3′
	5′-GAAGATGGTGATGGGATTTC-3′

### Western blot analysis

After transfection for 48 h, a total of 1 × 10^6^ cells from each group were detected using Western blot analysis for expressions of ezrin, apoptosis-related proteins (including Bax, Bcl-2, p21, p53, and Caspase-3), and invasion-related proteins (including MMP-2 and MMP-9). The collected cells were washed with PBS for two to three times, added with SDS/PAGE sample buffer (1 × 10^7^/ml), boiled for 10 min for SDS/PAGE electrophoresis, and proteins with different molecular weights were separated. The gel was transferred on to nitrocellulose membranes, blocked with 5% skim milk for 1 h, and incubated with antibodies including rabbit anti-human ezrin (1:250, ab41672), rabbit anti-human Bax (1:1000, ab10813), rabbit anti-human Bcl-2 (1:500, ab59348), rabbit anti-human p21 (1:500, ab47452), rabbit anti-human p53 (1:500, ab31333), rabbit anti-human Caspase-3 (1:500, ab44976), rabbit anti-human MMP-2 (1:500, ab37150), rabbit anti-human MMP-9 (1:1000, ab38898), rabbit anti-human Cyclin D1 (1:1000, ab134175), and rabbit anti-human cyclin-dependent kinase 4a (CDK4a, 1:1000, ab108357) at 37°C for 1 h. Then the samples were washed with TBS with Tween-20 (TBST) (10 Mm Tris, pH 7.5, HCl, 100 Mm NaCl, and 1 g/l Tween-20). Second antibody goat anti-rabbit IgG marked by horseradish peroxidase (HRP) was added to the washed membranes and reacted for 2 h, and then washed with TBST buffer for four times. The membranes were scanned using ECL and photographed using gel imaging system (Bio-Rad Laboratories, Hercules, CA, U.S.A.) for gray-scale value analysis. The rabbit anti-human polyclonal antibodies (ezrin, Bax, Caspase-3, Bcl-2, MMP-2 and MMP-9) were purchased from Abcam Inc., (Cambridge, MA, U.S.A.). The experiment was repeated three times.

### ELISA

After transfection for 48 h, the culture medium (200 μl) was extracted and MG-63 cells were centrifuged with cell supernatant obtained. Next, the standard product was prepared according to the instructions of ELISA kit (PH029RAT, Phygene, Fuzhou, Fujian, China), and the levels of Caspase-3, MMP-2 and MMP -9 were determined. After loading, cells were incubated at 37°C for 60 min, and added with 100 μl enzyme-labeled antibody. The plates were washed with 0.01 M pH 7.4 poly(butylene succinate-co-butylene terephthalate) (PBST) five times, and cells were developed with chromogenic agent avoiding light for 10 min. The optical density (OD) value at 450 nm was measured, and the concentrations of Caspase-3, MMP-2 and MMP-9 were calculated. The experiment was repeated three times.

### MTT assay

After transfection for 48 h, 1 × 10^4^ cells from each group were placed into a 96-well plate and cultured under conditions of 37°C, 5% CO_2_ for 24, 48, 72, and 96 h, respectively. A total of 10 μl MTT solution (5 mg/ml) (Sigma Company, St. Louis, MO, U.S.A.) was added to each well and then the cells were continued to be cultured under conditions of 37°C, 5% CO_2_ for 4 h. The reaction was terminated with the addition of 10% SDS-HCl solution (100 μl) to each well. Subsequently, the OD value at 570 nm was measured using a microplate reader (Bio-Rad Laboratories, Hercules, CA, U.S.A.) after the solution was fully dissolved. Growth curves were drawn with time in x-axis and OD value in y-axis to calculate growth inhibition rate (GIR) of cells. GIR = [(OD_control group_ − OD_test group_)/OD_control group_] × 100%.

### Colony formation assay

The human osteosarcoma MG-63 cells at logarithmic growth phase were dissociated into single cell suspension till each cell reached 95% confluence. Next, 3 × 10^2^ cells were seeded in a six-well plate and transfected by the methods mentioned above with three replicates in each group. The plate was gently shaken to obtain a homogeneous mixture. The cells were cultured under conditions of 37°C, 5% CO_2_ for 18 days, with culture fluid changed once every 3 days. The culture was ended until the clone spots were visible to naked eyes. The cells were washed with PBS for two to three times after the removal of culture fluid. Glutaraldehyde (5%) was added into the cells for fixation for 5 min after drying at room temperature. Then the cells were added with 0.1% Crystal Violet (500 μl) to stain for 10 min, followed by washing with PBS, and then calculated under the microscope after drying at room temperature.

### Flow cytometry

After transfection for 48 h, 1 × 10^6^ cells in each group were cultured with 5% CO_2_ at 37°C for 48 h. The cells were collected after centrifugation at 2000 rpm for 5 min. Then the cells were collected again after mixing with 1 ml PBS without the supernatant, for two circles. With 1× binding buffer to adjust the cell density to 1 × 10^6^ cells/ml, 100 μl cells were added to the flow tube, mixed with 10 μl Annexin-V-PE on ice, and reacted avoiding light for 15 min, then added with 380 μL of 1× binding buffer and 10 μl 7-ADD. The cells were detected using Epics-XL II flow cytometry (Beckman Coulter, Inc., Fullerton, CA, U.S.A.), and the number of apoptotic cells was calculated using CellQuest software by collecting 10000 cells. Apoptotic peak (Ap peak, G_1_ subpeak before G_1_) and cell circle were analyzed using ModFit software. The experiment was repeated three times.

### Transwell assay

Matrigel (Sigma Company, St. Louis, U.S.A.) were diluted by serum-free medium (1:3) on ice, and 100 μl was added to the upper chamber of the Transwell to establish the basement membrane. The human osteosarcoma MG-63 cells at logarithmic growth phase were transfected by the methods mentioned above for 48 h. After digestion with trypsin, the cells were washed with serum-free medium for three times, re-suspended by PBS. The concentration of the cell was adjusted to 1 × 10^5^ cells/ml. A total of 200 μl cells were added to the upper chamber and 500 μl FBS (20%) to the down chamber of the Transwell, with three replicates for each group. The cells were cultured under conditions of 37°C, 5% CO_2_ for 24 h. Then, the Transwell chambers were washed with PBS for twice, fixed with 5% glutaraldehyde for 5 min, and added with 0.1% Crystal Violet (500 μl). After 10-min staining, the chambers were washed with PBS and the inner cells on microporous membrane were dried. The cells were calculated and photographed under the microscope.

### Scratch test

The serum-free medium was mixed well with matrigel at a ratio of 9:1, then added into a six-well plate with 300 μl in each well and air-dried in refrigerator at 4°C. The cells were cultured under conditions of 37°C, 5% CO_2_ for 1 h with the addition of serum-free DMEM (containing 10 mg/ml BSA; Gibco, Life Technologies, Darmstadt, Germany). The human osteosarcoma MG-63 cells at logarithmic growth phase were assigned into three groups by the methods mentioned above, and seeded into a six-well plate (1 × 10^4^ cells/well). After cell adhesion, a straight line was drawn on the cell surface using a 20 μl sterile pipette tip along the straightedge. After the addition of the culture medium with full serum, the cells were cultured at 37°C with 5% CO_2_ for 24, 48, 72, and 96 h, respectively, and photographed under the microscope. Cell migration distance was calculated.

### Statistical analysis

Statistical analysis was conducted by using SPSS 19.0 (IBM Corp., Armonk, NY, U.S.A.). Student’s *t* test was applied for the comparisons between two groups, and comparisons amongst multiple groups were analyzed by one-way ANOVA. A *P*-value <0.05 was considered to be statistically significant.

## Results

### The ezrin 2 siRNA sequence is used for recombinant adenovirus expression vector of ezrin

**Figure 1:**
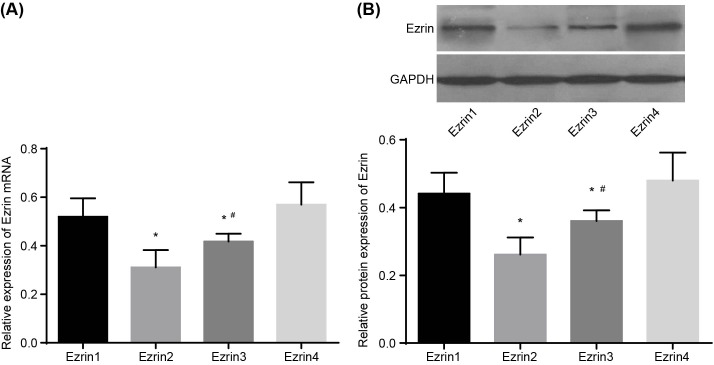
RT-qPCR and Western blot analysis show that ezrin 2 siRNA sequence is employed for recombinant adenovirus expression vector of ezrin (**A**) Relative mRNA expression of ezrin 1 and 4 are higher than that of ezrin 2 and ezrin 3 detected by RT-qPCR. (**B**) Relative protein levels of ezrin 1 and 4 are higher than that of ezrin 2 and ezrin 3 detected by Western blot analysis; *, *P*<0.05 compared with ezrin 1 and ezrin 4; ^#^, *P*<0.05 compared with ezrin 2.

**Figure 2:**
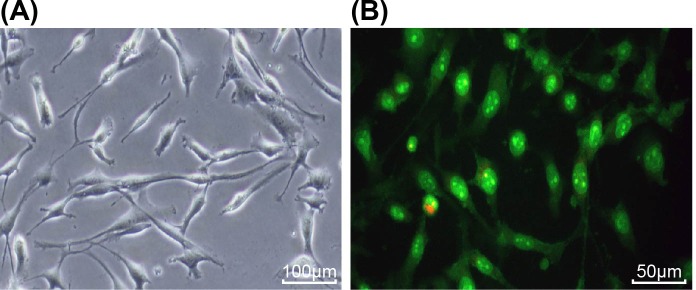
MG-63 cells transfected with adenovirus-mediated ezrin recombinant plasmids display green fluorescence after G418 selection and amplification (×200) (**A**) Brightfield image of MG-63 cells infected with Ad-Ezrin recombinant adenovirus vector; (**B**) fluorography of MG-63 cells infected with Ad-Ezrin recombinant adenovirus vector

RT-qPCR and Western blot analysis were adopted to determine the expression levels of ezrin 1, ezrin 2, ezrin 3, and ezrin 4. The results showed that compared with ezrin 1 and ezrin 4, down-regulation of ezrin 2 and ezrin 3 siRNA was significant (*P*<0.05), in which the down-regulation of ezrin 2 siRNA was more apparent than ezrin 3 siRNA (*P*<0.05), thus the ezrin 2 siRNA sequence was employed for the recombinant adenovirus expression vector of ezrin (Figure 1). After G418 selection, MG-63 cells that were transfected with Ad-Ezrin recombinant adenovirus plasmids gave out green fluorescence (Figure 2), and after multiple rounds of transfection, the cells reached a high titer of 10^8^ pfu/ml.

### Adenovirus-mediated siRNA targetting ezrin regulates expression of ezrin in MG-63 cells

**Figure 3:**
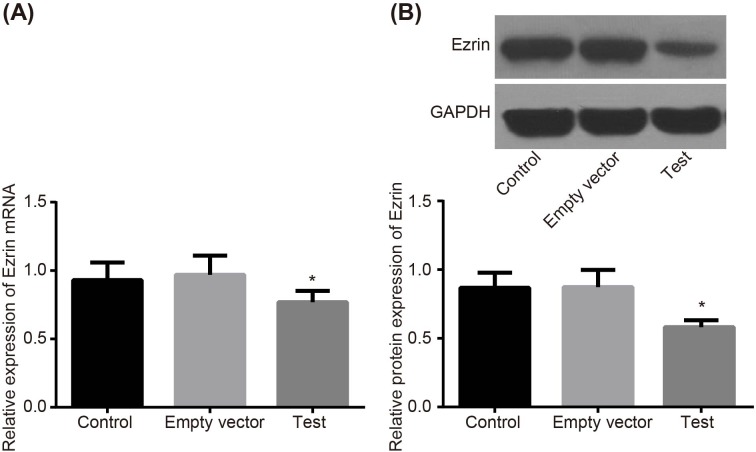
RT-qPCR and Western blot analysis show that adenovirus-mediated siRNA targetting ezrin reduces expression of ezrin in MG-63 cells (**A**) mRNA expression of ezrin detected by RT-qPCR. (**B**) Protein level and protein band pattern of ezrin detected by Western blot analysis; *, *P*<0.05 compared with the control group.

RT-qPCR and Western blot analysis were performed to investigate the effects of adenovirus-mediated siRNA targetting ezrin on expression of ezrin in MG-63 cells. The results showed that there was a weak specific band ~431 bp in the test group, while in the control and empty vector groups the band was obvious. The results of Western blot analysis were consistent with that of RT-qPCR, which indicated that the recombinant adenovirus vector could mediate the mRNA and protein levels of ezrin in MG-63 cells (Figure 3).

### Adenovirus-mediated siRNA targetting ezrin inhibits viability but induces apoptosis of MG-63 cells

**Figure 4:**
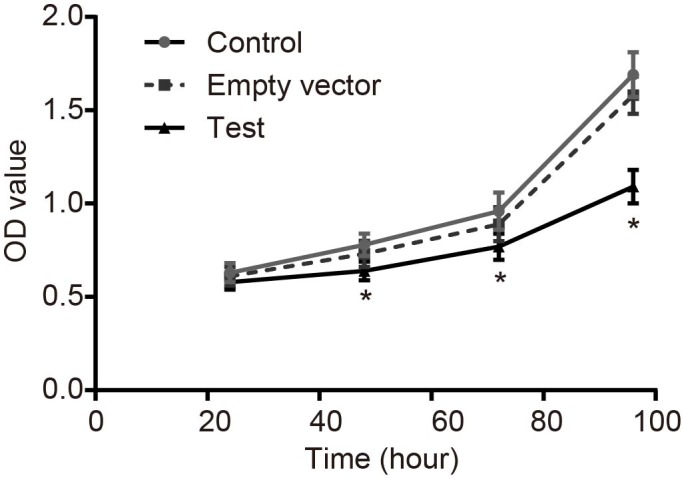
MTT assay shows that adenovirus-mediated siRNA targetting ezrin inhibits viability of human osteosarcoma MG-63 cells *, *P*<0.05 compared with the control group.

**Figure 5:**
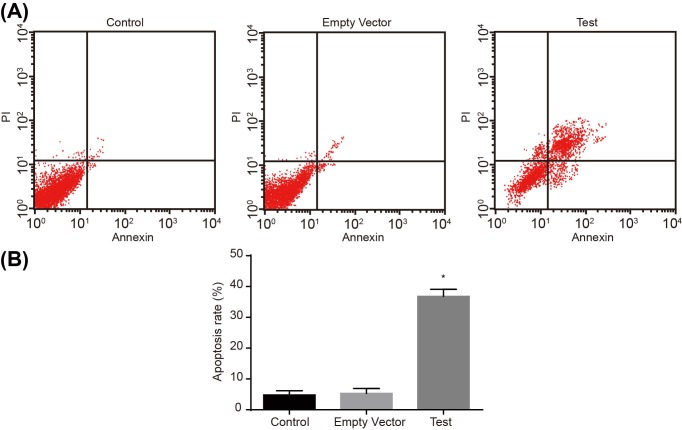
Flow cytometry shows that adenovirus-mediated siRNA targeTting ezrin induces apoptosis rate of human osteosarcoma MG-63 cells (**A**,**B**) Adenovirus-mediated siRNA targetting ezrin increased cell apoptosis. *, *P*<0.05 compared with the control group. Abbreviation: 7-AAD, 7-aminoactinomycin D.

**Figure 6:**
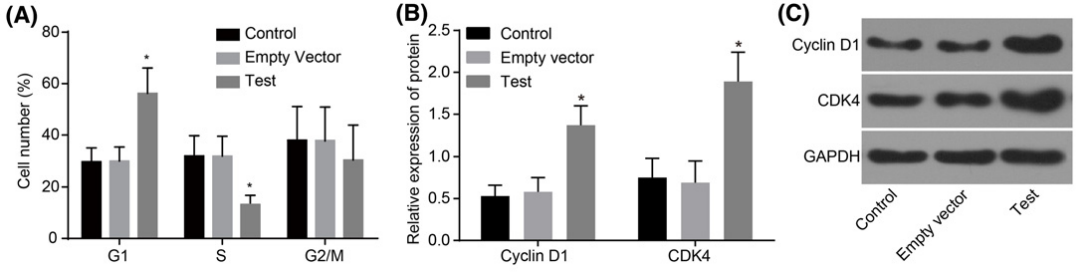
Western blot analysis shows that adenovirus-mediated siRNA targetting ezrin elevates protein levels of Cyclin D1 and CDK4a in human osteosarcoma MG-63 cells (**A**) Cell cycle entry detected by flow cytometry; (**B**), protein levels of Cyclin D1 and CDK4a detected by Western blot analysis. (**C**) Protein band patterns of Cyclin D1 and CDK4a detected by Western blot analysis; *, *P*<0.05 compared with the control group.

**Figure 7:**
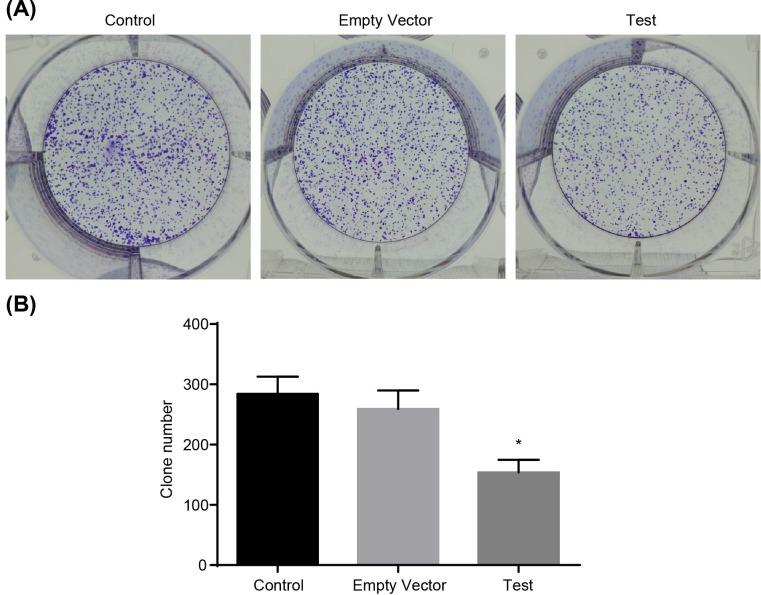
Colony-formation assay shows that adenovirus-mediated siRNA targetting ezrin inhibits growth of human osteosarcoma MG-63 cells. (**A**) morphology of cloned cells; (**B**) number of cloned cells. *, *P*<0.05 compared with the control group

MTT assay was adopted to detect cell viability. As shown in Figure 4, no significant differences were found in the three groups at 24 h (all *P*>0.05). While at 48 h, compared with the control and empty vector groups, the growth of MG-63 cells in the test group was obviously inhibited and time dependent (*P*<0.05). Meanwhile, no significant difference was observed between the control group and the empty vector group at any time (*P*>0.05). Flow cytometry was used to detect the apoptosis rate of MG-63 cells at 48 h after transfection. The results showed that the apoptotic rate in the control group was 4.8%, and compared with the control group, apoptosis of MG-63 cells in the test group was significantly induced, which was 36.8% (*P*<0.05), while the apoptotic rate in the empty vector group was 5.3%, which showed no significant difference (*P*>0.05) (Figure 5). The cell cycle of MG-63 was detected by flow cytometry 48 h after transfection and the results demonstrated that, compared with the control and empty vector groups, cells in the test group increased significantly at G_1_ phase, and decreased distinctly at S-phase (both *P*<0.05). There was no significant difference between the control and empty vector groups (*P*>0.05) (Figure 6A). Western blot analysis was performed to detect the protein levels of Cyclin D1 and CDK4a. Compared with the control and empty vector groups, the test group displayed obviously elevated protein levels of Cyclin D1 and CDK4a (all *P*<0.05). There was no significant difference between the control and empty vector groups (*P*>0.05) (Figure 6B,C). Cells in each group were conducted with colony formation assay and no significant difference was observed between the control group and the empty vector group (*P*>0.05). Cloned cells in the test group were obviously less than in the control and empty vector groups (both *P*<0.05) (Figure 7). These findings indicated that adenovirus-mediated siRNA targetting ezrin inhibits viability but induces apoptosis of MG-63 cells.

### Adenovirus-mediated siRNA targetting ezrin inhibits MG-63 cell invasion and migration

**Figure 8:**
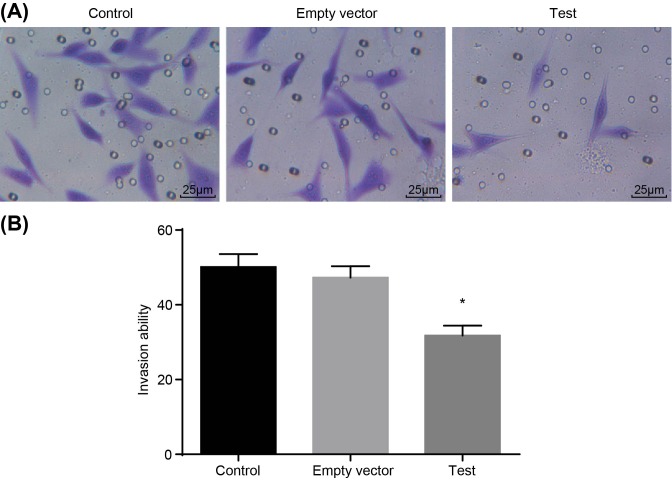
Transwell assay shows that adenovirus-mediated siRNA targetting ezrin inhibits invasion of human osteosarcoma MG-63 cells (**A**) morphology of of invasive cells; (**B**) cell invasion ablilities. *, *P*<0.05 compared with the control group.

**Figure 9:**
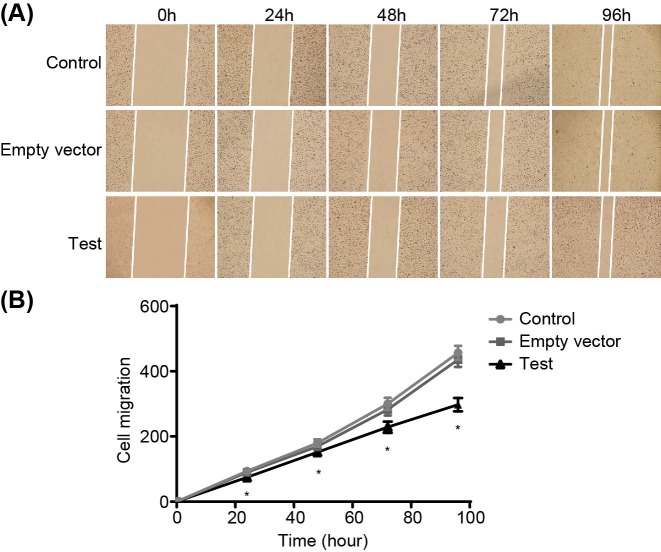
Scratch test shows that adenovirus-mediated siRNA targetting ezrin inhibits migration of human osteosarcoma MG-63 cells (**A**) morphology of migrated cells; (**B**) cell migration abilities. *P<0.05 compared with the control group.

MG-63 cell invasion and migration were detected by Scratch test and Transwell assay. The ability of osteosarcoma cells to pass through the basement membrane formed by Matrigel reflects its invasion ability. Compared with the control group and the empty vector group, the invasion and migration of human osteosarcoma MG-63 cells in the test group was significantly decreased (both *P*<0.05), while no palpable difference was found in the invasion and migration of cells in the control group and the empty vector group (*P*>0.05). All results indicated that si-ezrin inhibit the invasion and migration of human osteosarcoma MG-63 cells (Figures 8 and 9).

### Adenovirus-mediated siRNA targetting ezrin elevates expression levels of Bax, p21, p53, and Caspase-3, and reduces expression levels of Bcl-2, MMP-2 and MMP-9

**Figure 10:**
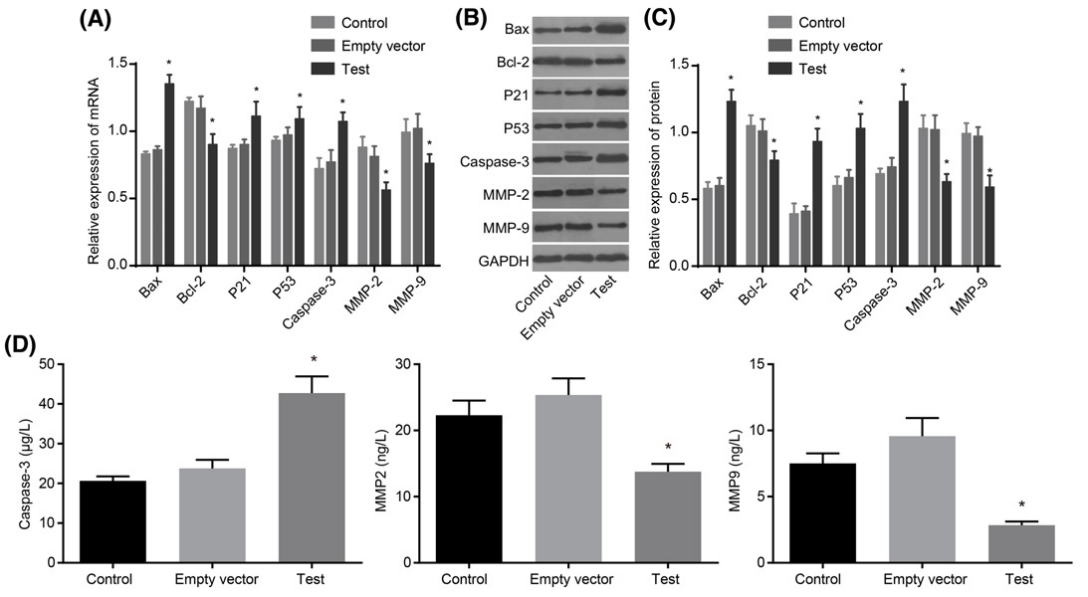
RT-qPCR, Western blot analysis, and ELISA show that adenovirus-mediated siRNA targetting ezrin inhibits cell apoptosis and invasion in human osteosarcoma MG-63 cells (**A**) mRNA expressions of Bax, Bcl-2, p21, p53, Caspase-3, MMP-2 and MMP-9 detected by RT-qPCR. (**B**) Protein band patterns of Bax, Bcl-2, p21, p53, Caspase-3, MMP-2 and MMP-9 detected by Western blot analysis. (**C**) Protein levels of Bax, Bcl-2, p21, p53, Caspase-3, MMP-2 and MMP-9 detected by Western blot analysis. (**D**) Levels of Caspase-3, MMP-2 and MMP-9 detected by ELISA; *, *P*<0.05 compared with the control group.

RT-qPCR and Western blot analyses were adopted to determine the expression levels of apoptosis- and invasion-related genes. Compared with the control and empty vector groups, expression levels of Bax, p21, p53, and Caspase-3 in MG-63 cells significantly increased in the test group, and expression levels of Bcl-2, MMP-2, and MMP-9 obviously decreased (all *P*<0.05). There was no significant difference in expression levels of these genes between the control and empty vector groups (all *P*>0.05) (Figure 10A–C).

ELISA was adopted to further measure the levels of Caspase-3, MMP-2 and MMP-9. The results showed that, compared with the control and empty vector groups, Caspase-3 expression level in MG-63 cells in the test group significantly increased, while expression levels of MMP-2 and MMP-9 obviously decreased (all *P*<0.05). There was no significant difference in expression levels of Caspase-3, MMP-2 and MMP-9 between the control and empty vector groups (all *P*>0.05) (Figure 10D). These findings indicated that adenovirus-mediated siRNA targetting ezrin elevates expression levels of Bax, p21, p53, and Caspase-3, and reduces expression levels of Bcl-2, MMP-2 and MMP-9.

## Discussion

The present study aims to investigate the effect of recombinant adenovirus mediated si-ezrin on the proliferation, migration, invasion, and apoptosis of human osteosarcoma MG-63 cells. The findings of our tests revealed that adenovirus-mediated siRNA targetting ezrin can inhibit the proliferation, migration, and invasion of MG-63 cells, and induce apoptosis of MG-63 cells, which has certain reference value in the gene therapy for the metastasis of human osteosarcoma.

Initially, our study found that adenovirus-mediated siRNA targetting ezrin can mediate the mRNA and protein expressions of ezrin in human osteosarcoma MG-63 cells. As one widely studied cytoskeleton linker protein, ezrin was found widely expressed in the cytoplasm, and previous study also demonstrated that the ezrin expression was related with the growth, metastasis, and poor prognosis in osteosarcoma [19,20]. Ezrin plays its role mainly in connecting physically and functionally the actin cytoskeleton with the cell membrane, which makes it essential for multiple fundamental cellular processes, such as cell adhesion, motility, and membrane transport with signaling pathways, and is found to be activated in tumors, especially with early metastasis [21,22]. Besides, Celik et al. [23] have proved that high expression of ezrin associated with metastasis and poor outcome in osteosarcoma. Adenoviruses have been widely studied and continually used as non-enveloped DNA viruses for cancer gene therapy research [24]. Adenoviruses were good at safety profile and their ability to achieve a high level of transgene expression in the treatment for various tumor diseases, which contributes to the progress of gene therapy associated with malignant glioma [25]. However, they are limited by inefficient adenovirus vector mediated gene transfer and poor conversion in tumor tissues, and sometimes adenovirus can result in both transgene expression and acute augmentation of virus particles, which may cause hepatotoxicity [26,27]. Furthermore, it was proved that as an oncolytic adenoviral vector, organic anion transporter (OAT) is a promising and efficient expression vector for osteosarcoma gene therapy, which can efficiently and selectively replicate osteosarcoma cells to up-regulate the expression of related transferred genes [28].

In addition, it was also found that adenovirus-mediated siRNA targetting ezrin inhibits the proliferation, migration, and invasion of MG-63 cells, impedes cell cycle progression and induces apoptosis of MG-63 cells. As a cross-linker between the plasma membrane and actin cytoskeleton, ezrin participated in cell motility, membrane trafficking, as well as cell adhesion and apoptosis [29]. Bulut et al. [30] demonstrated that ezrin can inhibit invasion by small molecule inhibitors to osteosarcoma cells. And Zhao et al. [31] had demonstrated that *miR-183* repressed the expression of ezrin and significantly inhibited the motility and invasion of osteosarcoma cells. It has been reported that high ezrin expression was associated with metastasis and poor outcome in pediatric patients with osteosarcoma [21,32]. Furthermore, our study also demonstrated that MG-63 cells in the test group had up-regulated mRNA and protein expressions of Bax, p21, p53, and Caspase-3 and down-regulated mRNA and protein expressions of Bcl-2, MMP-2 and MMP-9. As a pro-apoptotic member of Bcl-2 family, Bax exits from the cytosol to mitochondria, where it permeabilizes and oligomerizes the mitochondrial outer membrane thus to promote apoptosis [33,34]. The p53 is involved in regulating the development of negative cellular as a tumor suppressor protein, while p21 belongs to p53 transcription targets which functions as a tumor suppressor including the apoptosis and arrests cell cycle as well as its inhibitory activity in cell cycle [35,36]. Moreover, Cyclin D1, the allosteric regulator of CDK4, is an integral regulator of growth factor-dependent G_1_-phase progression, and CDK4 is also involved in progression through the G_1_–S phases [37,38]. As a group of proteases were instrumental, caspases were involved in many cellular functions such as cell remodeling, differentiation, and death, in particularly, Caspase-3 is not only very important in neuronal apoptosis but also regarded as the terminal event before cell death [39]. MMP-2 and MMP-9 are both secreted, cancer-associated, zinc-dependent endopeptidases, which play key roles in regulation of some crucial signaling pathways in cell growth, invasion, migration, angiogenesis, and inflammation [40]. Consistently, it has been proved that adenoviral vector-mediated IL-24 expression suppresses the growth of MG-63 osteosarcoma cells through down-regulating Bcl-2 expression, and up-regulating Bax and Caspase-3 expressions [41,42].

In conclusion, our observations demonstrated that adenovirus-mediated siRNA targetting ezrin can inhibit the proliferation, migration, and invasion of MG-63 cells, and induce apoptosis of MG-63 cells, which may be clinically helpful to cancer gene therapy for osteosarcoma treatment. Further studies with more detailed data are needed to provide deep investigation about the specific effects.

## Abbreviations

Bax: Bcl2-associated X protein
Bcl-2: B cell lymphoma-2
CDK4a: cyclin-dependent kinase 4a
DEPC: diethyl pyrocarbonate
DMEM: Dulbecco’s modified Eagle’s medium
GAPDH: glyceraldehyde-3-phosphate dehydrogenase
GIR: growth inhibition rate
IL-24: interleukin-24
MMP: matrix metalloproteinase
OD: optical density
PBST: poly(butylene succinate-co-butylene terephthalate)
pfu: plaque-forming unit
RT-qPCR: reverse-transcription quantitative PCR
TBST: TBS with Tween-20
